# Abnormal Gait Phase Recognition and Limb Angle Prediction in Lower-Limb Exoskeletons

**DOI:** 10.3390/biomimetics10090623

**Published:** 2025-09-16

**Authors:** Sheng Wang, Chunjie Chen, Xiaojun Wu

**Affiliations:** 1 School of Mechanical and Electrical Engineering, Xi’an University of Architecture and Technology, Xi’an 710055, China; 2 Shenzhen Institutes of Advanced Technology, Chinese Academy of Sciences, Shenzhen 518055, China

**Keywords:** Convolutional Neural Network, gait phase/stage, lower-limb exoskeleton, long short-term memory neural network, motion prediction

## Abstract

The phase detection of abnormal gait and the prediction of lower-limb angles are key challenges in controlling lower-limb exoskeletons. This study simulated three types of abnormal gaits: scissor gait, foot-drop gait, and staggering gait. To enhance the recognition capability for abnormal gait phases, a four-discrete-phase division for a single leg is proposed: pre-swing, swing, swing termination, and stance phases. The four phases of both legs further constitute four stages of walking. Using the Euler angles of the ankle joints as inputs, the capabilities of a Convolutional Neural Network and a Support Vector Machine in recognizing discrete gait phases are verified. Based on these discrete gait phases, a continuous phase estimation is further performed using an adaptive frequency oscillator. For predicting the lower-limb motion angle, this study innovatively proposes an input scheme that integrates three-axis ankle joint angles and continuous gait phases. Comparative experiments confirmed that this information fusion scheme improved the limb angle prediction accuracy, with the Convolutional Neural Network–Long Short-Term Memory network yielding the best prediction results.

## 1. Introduction

Exoskeletons, whose core design philosophy originates from the imitation of biological motion systems, can achieve human capability enhancement, motor function compensation, and medical rehabilitation through bionic structures, biomimetic materials, and biologically inspired motion mechanisms. A typical lower-limb exoskeleton (LLE) generally employs sensors to detect the user’s gait phase within a gait cycle, carrying out motion prediction and planning according to the gait phase, and enabling locomotion assistance via actuators. The gait phase detection and the motion prediction are important controlling components in the LLE systems [1].

The Gait phases can provide appropriate control parameters and strategies for the LLEs across different stages of the user’s walking, ensuring assistance aligns with natural human movement. Numerous studies have proposed discrete phase division schemes, such as 4-phase [2,3], 5-phase [4], and 6-phase [5]. There are mainly two types of classifiers for discrete gait detection. One is the threshold-based method. For example, pressure thresholds or movement signal thresholds of the foot can be used to detect each gait phase [3,6,7,8]. Moreover, signal thresholds from other body parts can also be applied to realize gait phase detection [5]. The other type of phase detection is a classifier based on machine learning and deep learning. Common classifiers include Hidden Markov models [3], support vector machines (SVMs) [4], Various neural networks, such as Long Short-Term Memory (LSTM) Networks [9], and Convolutional Neural Networks (CNNs) [10]. Discrete gait phases are difficult to express all motion states, and a smooth transition between discrete states is difficult to achieve. Therefore, many studies have carried out research on continuous phase estimation. Continuous phase methods demonstrate superior capability over discrete gait approaches in reproducing biologically authentic locomotion patterns. An example of continuous phase estimation is a time-based estimator. This method uses the historical gait cycle as a reference and obtains the percentage value of the current phase by the ratio of the current gait duration to the historical gait cycle. This method is easy to operate, but it cannot adapt to the situation where the gait rhythm changes. The adaptive frequency oscillator (AFO) is another commonly used continuous phase estimation method [11,12], which can estimate the gait cycle according to the frequency characteristics of a certain event without prior knowledge, and it is particularly suitable for situations where the walking frequency changes. However, from a perspective of practical applications, the AFO’s ability to adapt to complex situations is still limited. Another commonly used method for continuous phase estimation is based on the Extended Kalman Filter (EKF) [13]. Compared to the AFOs that enforce phase synchronization via designed nonlinearities, the EKF estimates phase as an unobserved state variable through stochastic filtering of noisy measurements, though its performance is limited by model inaccuracies, noise sensitivity, and computational complexity. In addition, machine learning and neural networks are also applied in continuous phase estimation [14].

The motion state prediction of LLEs helps prevent the time delay of control strategies. A practice easy to implement is that LLEs adopt fixed, pre-defined trajectories, where the LLE leads and the human follows. This method is mostly applied to users with severely impaired motor function. However, the motion trajectories based on fixed pre-defined trajectories struggle to align with the natural human gait, often causing discomfort to users. Therefore, for users with mobility capabilities, real-time prediction of how LLEs adapt to human movements has become crucial. One effective approach is dynamic movement primitives (DMPs) [15,16], which enable online trajectory generation by parameterizing movement patterns through a nonlinear dynamical system. DMPs suffer from weak trajectory generalization ability, poor adaptability to complex environments, experience-dependent parameter adjustment, and complex high-dimensional motion modeling, making it difficult to meet dynamic motion requirements. The central pattern generator (CPG) is another relatively common means for predicting the LLE movement [17,18]. The CPG, inspired by biological neural rhythm principles, constructs coupled oscillator systems via mathematical models to autonomously generate rhythmic signals. CPGs are constrained by inaccurate mapping of motor intentions, excessive simplification of biological models, poor robustness in real-time feedback adjustment, and non-smooth multi-mode switching, which hinder the realization of high-precision motion control. Additionally, machine learning or deep learning, as a data-driven method, can be applied to both users with motor ability (for motion prediction) and those without (for trajectory generation). Examples include Radial Basis Function networks [19], LSTM networks [20], and Generalized Regression networks [21]. Although machine learning or deep learning methods are widely used in the LLE control, these methods require a large volume of data for training, and the generalization ability of the models still needs to be further enhanced.

In essence, the academic community has conducted extensive research on gait phases and motion prediction in the field of LLEs. The overarching objective of these studies is to enable the LLEs to closely mimic natural human motion, thereby meeting critical requirements for comfort, safety, and practical utility. However, existing gait studies still have some shortcomings: (1) Most gait research is oriented toward normal populations, with insufficient personalized adaptation for abnormal gaits. Individuals with abnormal gaits have weak mobility, making it difficult to walk continuously. Their gaits lack periodicity, and the movements of the left and right legs lack symmetry, resulting in more complex and diverse forms of abnormal gaits, such as scissor gait, foot-drop gait, and staggering gait. For the same type of abnormal gait, there are often large differences because of different injury degrees and rehabilitation processes of patients. Therefore, applying the gait phase division and detection methods for normal populations to abnormal gaits often fails to achieve good control effects. (2) The motion prediction models adapt poorly to data from abnormal gaits. The common prediction models include the CNNs, which are suitable for spatial data and the Recurrent Neural Networks and their variant LSTM neural networks, which are more adapted to time-series data. The gait data contain both spatial and temporal features, with strong coupling between the two. However, the existing data processing methods often separate spatial and temporal features, preventing models from learning such coupling relationships and thus impairing the accuracy of predictions. The temporal patterns of abnormal gaits exhibit high variability, making it difficult for prediction models to capture long-range correlations across cycles.

These challenges have severely restricted the application of the LLEs in the field of medical rehabilitation. This study argues that a “subject-specific” approach should be adopted for the recognition of abnormal gait phases and the lower-limb movements to adapt to the challenges posed by diverse users. In terms of specific implementation paths, a method for dividing gait into four discrete phases for the LLEs is proposed, which can be adapted to unilateral healthy gaits or roughly symmetric abnormal gaits. Three types of abnormal gaits were simulated, and the CNN and the SVM were employed to recognize the discrete phases of these three abnormal gaits. Based on discrete phases, a continuous phase estimation method based on an AFO was developed. Finally, in terms of the lower-limb prediction model, the three-axis angles of the ankle joints were combined with gait phases to achieve the fusion of spatial and temporal features, and the fused signals were used as the model inputs to predict the angles of the thighs and shanks in the sagittal plane (For the sake of narrative convenience, unless explicitly stated otherwise, all angle descriptions mentioned in the following text refer to the Euler angles in the sagittal plane). Comparative experiments were designed to verify the prediction capabilities of multiple neural networks, including the CNN, the LSTM, and their combined CNN_LSTM.

## 2. Division of Gait Phases

Analyzing continuous walking (both normal and abnormal gaits) reveals that the left and right limbs must coordinate. When one limb ends its movement, the other starts, and when one limb swings, the other remains in the stance phase. For the LLEs, assistance primarily occurs during the swing phase, while the mechanical and electrical systems remain inactive during the stance phase. Thus, from an assistive strategy perspective, only the swing-related phases need differentiation, rather than further refinement of the stance phase.

Based on this analysis, the human gait can be divided into four phases: pre-swing, swing, swing termination, and stance, labeled as 1, 2, 3, and 0, respectively. The four gait phases during walking exhibit the following characteristics:

- Pre-swing: The lower limb is at the rearmost position of the gait cycle, with the heel off until the toe off. The contralateral limb is in the swing termination phase 3. The LLE activates the drive mechanism on the ipsilateral side to prepare for assistance.
- Swing: The lower limb moves forward, with the foot completely off the ground. The contralateral limb is in the stance phase 0. The LLE drives the actuator on the ipsilateral side to provide active assistance.
- Swing termination: The lower limb is at the foremost position of the gait cycle, from heel-strike to foot-flat. The contralateral limb is in the pre-swing phase 1. The LLE resets the actuator on the ipsilateral side to its initial position.
- Stance: The foot fully contacts the ground, supporting the body’s weight. The contralateral limb is in the swing phase 2. The actuator on the ipsilateral side remains inactive.

**Figure 1:**
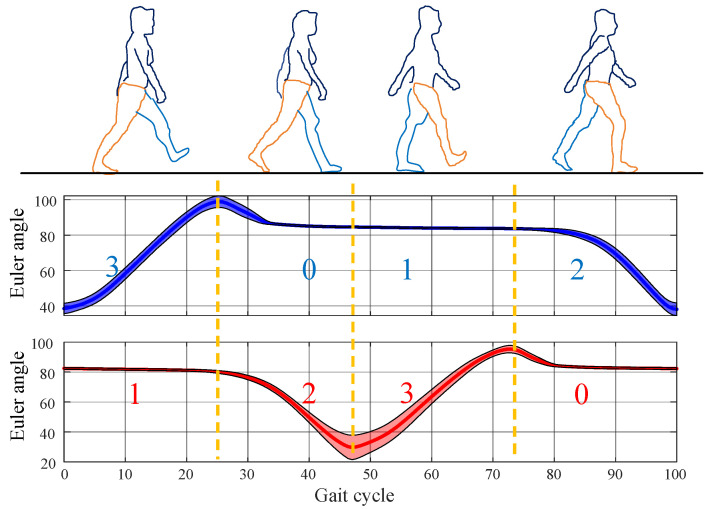
Four phases and corresponding ankle joint Euler angles. Blue represents the left ankle joint Euler angles, and red represents the right ankle joint Euler angles. The digits indicate the gait phases of the left and right feet, respectively.

The ankle joint provides rich motion information, where the sagittal Euler angles can determine various gait events through an intuitive trend and threshold judgments, facilitating phase determination. The proposed four-phase division and corresponding ankle joint Euler angles are shown in Figure 1:

The combination of the four phases of the left and right feet is referred to as the four stages. The four stages follow a cyclic pattern: left swing termination phase 3, right pre-swing phase 1 → left stance phase 0, right swing phase 2 → left pre-swing phase 1, right swing termination phase 3 → left swing phase 2, right stance phase 0. For clarity, these four stages are denoted as Gait 20, Gait 31, Gait 02, and Gait 13, where the first digit represents the left foot’s phase, and the second digit represents the right foot’s phase. The Euler angles significantly decrease in the pre-swing phase 1 and increase in the swing phase 2, which aids the phase detection. Due to the unique correspondence between left and right foot phases, determining the phase of one limb uniquely identifies that of the other. Thus, Gait 20 and Gait 02 are identified by detecting an upward trend in the Euler angles of the unilateral ankle joint. Gait 31 and Gait 13 are identified by detecting a significant downward trend in the Euler angles of the unilateral ankle joint. This classification method is particularly suitable for unilateral abnormal gait detection. The phase of the affected limb can be determined by verifying the gait of the healthy side.

## 3. Abnormal Gait Detection and Phase Estimation Algorithms

### 3.1. Gait Data Collection

This study selected three typical abnormal gaits as research objects: scissor gait, foot-drop gait, and staggering gait. Scissor gait is a roughly symmetric abnormal gait between left and right sides, while foot-drop and staggering gaits are unilateral abnormal gaits with the healthy side on the right and the affected side on the left.

Our experiment required repeated trials with the LLE, involving adjustments to control parameters. Patients with pathological conditions, if participating in the experiment, may face increased risks of fatigue, instability, or discomfort during prolonged testing. However, the focus of this study was to develop a control framework for LLEs that adapts to specific abnormal gait patterns, rather than investigating the underlying pathological mechanisms of these gaits. Therefore, we employed validated gait simulation protocols—which accurately replicated key clinical characteristics—as substitutes for actual pathological gaits to evaluate the LLE system’s capabilities in detecting, classifying, and responding to abnormal gait patterns. We recruited three healthy adults (all males; aged 24–28 years; height 175–182 cm) with no history of neuromuscular or locomotor disorders, who simulated the abnormal gaits. Prior to data collection, each participant received standardized training. They were shown clinical videos and biomechanical descriptions of each abnormal gait (e.g., scissor gait characterized by adducted thigh crossing during swing, foot-drop by insufficient dorsiflexion leading to toe drag, and staggering gait by irregular step timing and lateral trunk sway). A physical therapist specializing in gait disorders supervised practice sessions to ensure participants’ movements aligned with key features of the target pathological patterns.

**Figure 2:**
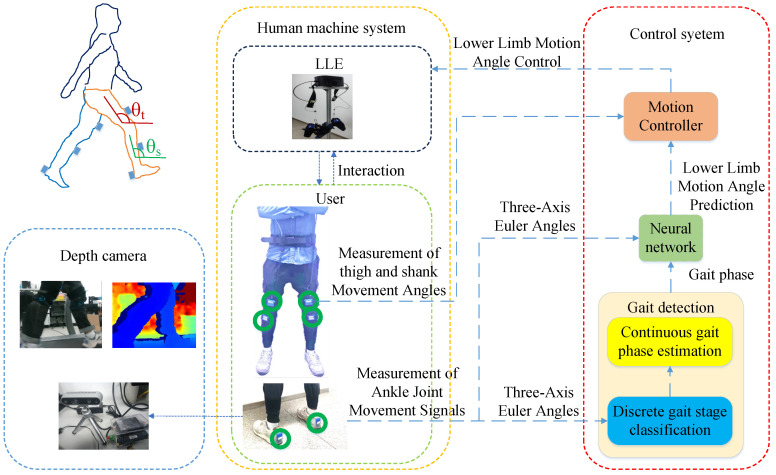
The HMI System. The motion sensors consist of six Inertial Measurement Units (IMU) [LPMS-B2, LP-Research Inc., Tokyo, Japan], each measuring Euler angle signals along three axes. The installation positions of the six motion sensors are marked with green circles. Additionally, an Intel RealSense D435i depth camera (68 × 17 × 7.25 mm^3^), which can capture RGB images and depth information images, assists in gait phase judgment. The three-axis Euler angles detected by the two IMUs at the heels are inputs to the gait phase estimator, which outputs phase information. The three-axis Euler angles are fused with the phase information and then used as inputs to the neural network, which outputs motion predictions for the lower limbs. The motion predictions of the lower limbs and the actual motion information fed back by the IMUs at the thighs and shanks are inputs to the motion controller to drive the LLE to work. *θ_t_* and *θ_s_* are the Euler angles of the thighs and shanks fed back by the IMUs.

Each participant wore an LLE an simulated walking with the three abnormal gaits in a laboratory for 3 min each. The participant and the LLE form a human-machine interaction (HMI) system (Figure 2), and the gait data were collected by motion sensors.

Collected gait data were manually labeled for phases by comparing ankle joint Euler angle trends with visual images from the depth camera. Twenty samples of gait cycles were extracted from each of the three types of abnormal gaits, and the “mean ± standard deviation” values were calculated to study the characteristics of abnormal gaits. Due to individual differences, fluctuations in movement status, and other factors, the number of data points in gait cycles varies among different samples. In this case, unifying the length through interpolation is a prerequisite for calculating the “mean ± standard deviation” values; otherwise, it is impossible to align the data point by point for statistical calculation. As shown in Figure 1, Euler angles of the same phase exhibit approximately linear changes, so the choice of linear interpolation is reasonable. Let two datasets be $A=\left[a_{1},a_{2},\ldots {,a}_{m}\right]$ and $B=\left[b_{1},b_{2},\ldots {,b}_{n}\right]$, where m>n. Taking the longest sample A as a reference, dataset B is extended to length m by determining interpolation points in the interval $\left[0,\;n-1\right]$ using Equation (1):

(1) $$x_{i}=\frac{i}{m-1}\times \left(n-1\right),\;i=0,\;1,\;\ldots ,\;m-1$$

where $x_{i}$ is the determined i-th interpolation point. Assuming that the interpolation point $x_{i}$ is between two known adjacent points ($x_{j},b_{j}$) and ($x_{j+1},b_{j+1}$), that is, $x_{j}\leq x_{i}\leq x_{j+1}$ is satisfied, the interpolation result $y_{i}$ of the interpolation point $x_{i}$ is determined according to Equation (2):

(2) $$y_{i}=b_{j}+\frac{b_{j+1}-b_{j}}{x_{j+1}-x_{j}}\times \left(x_{i}-x_{j}\right)$$

**Figure 3:**
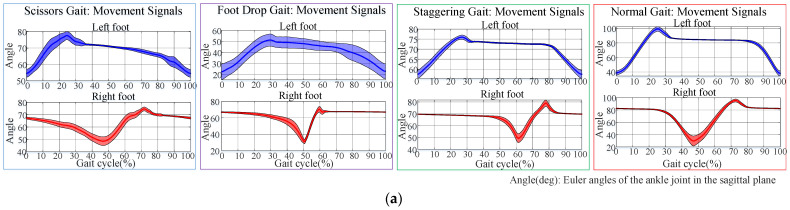

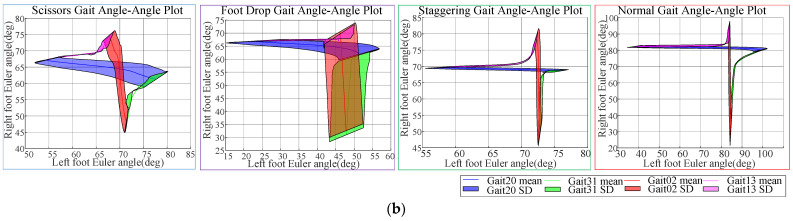
Comparison of ankle joint movement signals between abnormal gait and normal gait: (**a**) cycle–angle and (**b**) angle–angle.

After data alignment, a gait cycle contained 306 data points for scissor gait, 230 for foot-drop gait, and 198 for staggering gait. The data were normalized to the range of 0–100%. A comparison of the ankle joint Euler angles in the sagittal plane of each abnormal gait with normal gait is shown in Figure 3.

As shown in Figure 3, the Euler angles of the ankle joints in normal gait have a larger span and a smaller standard deviation, with smooth gait signals. For scissor gait, the boundary between the stance phases and pre-swing phases is not obvious, and the range of ankle Euler angle activity is narrow (between 50° and 80°). In foot-drop gait, the maximum value of ankle Euler angle is not significant, indicating the lack of heel strike action on the affected side. The stance phase duration is short, with the longest duration of the affected side’s stance phase being approximately 200 ms, the shortest 150 ms, and an average duration of 176 ms in samples. Meanwhile, the swing time of the affected side and the stance phase duration of the healthy side are longer, with the longest duration being approximately 700 ms, the shortest 350 ms, and an average duration of 515 ms, showing severe asymmetry between the left and right sides of the gait. In samples of staggering gait, the average difference between the maximum and minimum angles in the ascending segment is only 19.60°, while the average difference on the healthy side is 29.64°, indicating that the swing segment of staggering gait has a narrow range of activity, making this phase difficult to detect. Compared with the normal gait, all abnormal gaits lack smoothness in the Euler angle curve during the movement cycle and have larger standard deviations, indicating that the abnormal gaits lack stability during walking.

### 3.2. Identification of Abnormal Gait Stages

**Figure 4:**
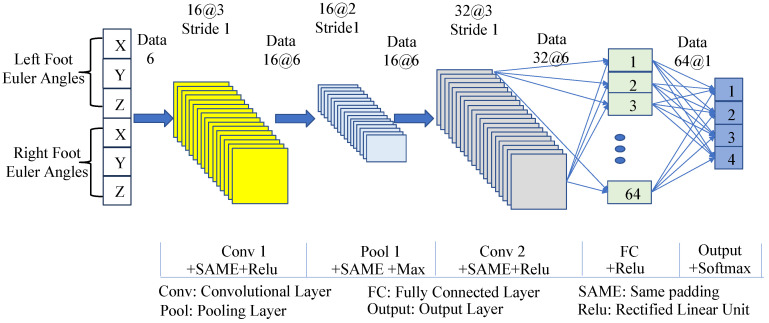
The structure of the CNN model for gait phase classification.

Despite the challenges in judging abnormal gaits, the superior classification performance of neural networks in nonlinear systems has made abnormal gait detection feasible. The CNN was used in this study to detect the discrete stages of three types of abnormal gaits, with the SVM being used for comparison. The classifier takes the Euler angles of the three axes of the left and right ankle joints (6 data points in total) as input and outputs four walking stages: Gait 20, Gait 31, Gait 02, and Gait 13, labeled as Class 1, Class 2, Class 3, and Class 4, respectively. The collected total samples included 4713 scissor gait samples, 3378 foot-drop gait samples, and 3135 staggering gait samples. For each abnormal gait, 60% of the samples were selected as the training set, 20% as the validation set, and 20% as the test set, with five-fold cross-validation adopted. The structure of the CNN used for gait stage detection is shown in Figure 4.

**Figure 5:**
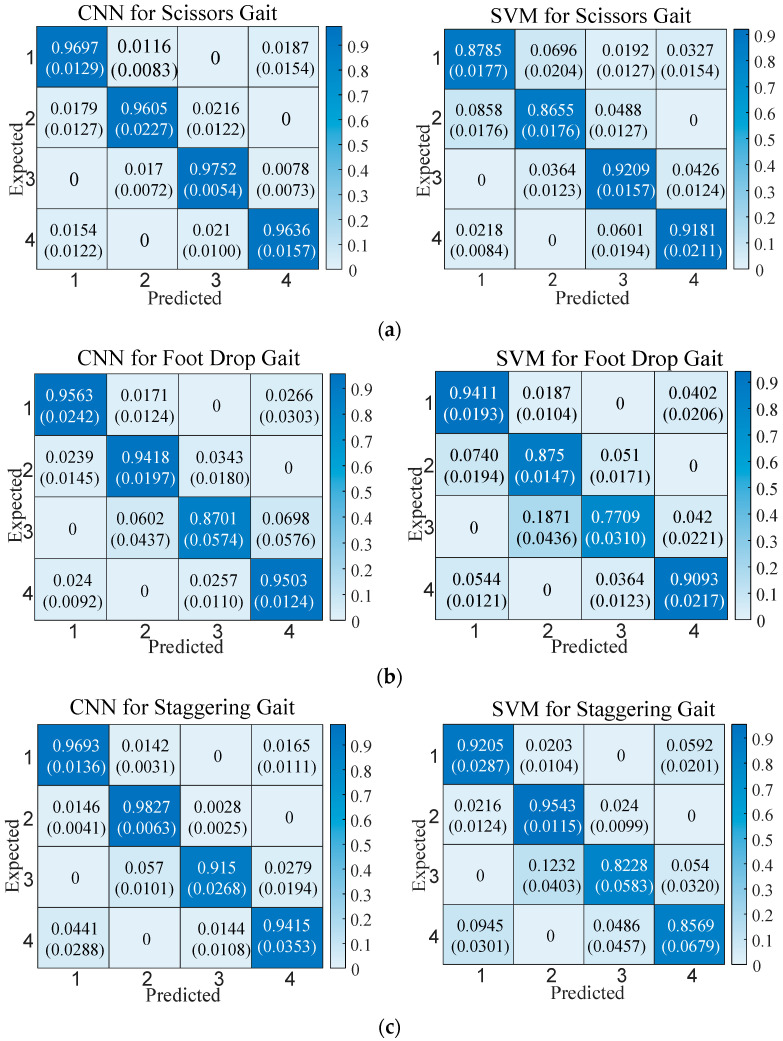
Classification accuracy of the CNN vs. the SVM. The top values show mean accuracy, and the bottom values (parentheses) show standard deviation. Recognition results of each stage of the (**a**) scissor gait, (**b**) foot-drop gait, and (**c**) staggering gait.

The convolutional layers and pooling layers of the CNN use the same padding to keep the output length of each layer consistent with the input. The convolutional layers and fully connected layers adopt Rectified Linear Unit (ReLU) activation functions, the pooling layer adopts max pooling, and the output layer uses the Softmax function. Other network settings include using Cross-Entropy Loss, a learning rate of 0.001, and 100 iterations. The support vector machine classifier uses a radial basis kernel function, and the regularization parameter (penalty factor) of the kernel function is 1. The recognition results of the two classifiers for abnormal gaits are shown in Figure 5.

In Figure 5, the CNN has higher classification performance than the SVM. The overall accuracy of the CNN for the three gaits can reach higher than 90%, while the overall accuracy of the SVM for the three gaits is lower than 90%. From the comparison of the classification results of each gait category, the CNN is also better than the SVM. An analysis of classification error causes for the two classifiers shows that almost all misclassifications occur during the transition phases of adjacent gaits. Only the SVM exhibits misclassifications in non-adjacent phases when classifying scissors gait.

To analyze the real-time performance of the two classifiers, the total time consumed by the two classifiers to recognize the test set samples was counted, as shown in Table 1.

In terms of time consumption comparison, the SVM has faster operational speeds than the CNN. However, both the CNN and the SVM can recognize a single gait phase at the microsecond level, and the classification time of the CNN can still meet real-time requirements. Therefore, considering both accuracy and real-time performance, the CNN is more conducive to gait phase classification.

From a control perspective, adopting continuous phases for LLE gaits has more advantages in achieving smooth control. Based on gait phase classification, this study converts each discrete state into a continuous phase value, ranging from 0 to 100%. Since the abnormal gaits do not have a stable period, time-based estimators (TBEs) are difficult to apply in such scenarios. However, the AFO can adapt to aperiodic gait characteristics by dynamically adjusting frequency, enabling accurate estimation of continuous phases. Therefore, the AFO was used in this study to achieve continuous phase estimation. The periodic input signal selected is the Euler angle of the left ankle joint. The periodic input $\theta \left(t\right)$ is reconstructed by a series of nonlinear periodic signals.

(3) $$\hat{\theta }\left(t\right)=\sum_{i=1}^{N}\alpha_{i}\left(t\right)\sin\left(\emptyset_{i}\left(t\right)\right)+\alpha_{0}\left(t\right)$$

where i is the index of the oscillators (i=1,2,…,N); $\alpha_{i}\left(t\right)$ is the amplitude of the oscillator; $\emptyset_{i}\left(t\right)$ is the phase of the oscillator; $\alpha_{0}\left(t\right)$ is the offset component of the oscillator; $\hat{\theta }\left(t\right)$ is the estimated result of the periodic input $\theta \left(t\right)$. The difference e(t) is calculated as the difference between the periodic input $\theta \left(t\right)$ and the estimate $\hat{\theta }\left(t\right)$, and this difference drives the dynamic evolution of state variables. The process is as follows:

(4) $${\dot{\emptyset }}_{i}(t)=\omega \left(t\right)\cdot i+\vartheta_{\emptyset }\frac{e\left(t\right)}{\sum \alpha_{i}}cos(\emptyset_{i}(t))$$

(5) $${\dot{\omega }}_{i}(t)=\vartheta_{\omega }\frac{e\left(t\right)}{\sum \alpha_{i}}cos(\emptyset_{i}(t))$$

(6) $${\dot{\alpha }}_{i}(t)=\eta e(t)sin(\emptyset_{i}(t))$$

(7) $${\dot{\alpha }}_{0}(t)=\eta e(t)$$

where ω is the fundamental frequency; $\vartheta_{\emptyset }$, $\vartheta_{\omega }$, and η are the constant gains determining learning speeds of the phase, frequency, and amplitude. The phase ${\dot{\emptyset }}_{1}(t)$ is used to estimate the continuous gait phase φ(t) as follows:

(8) $$\varphi (t)=\frac{mod({\dot{\emptyset }}_{1}(t),\;\;2\pi )}{2\pi }\times 100\%$$

**Figure 6:**
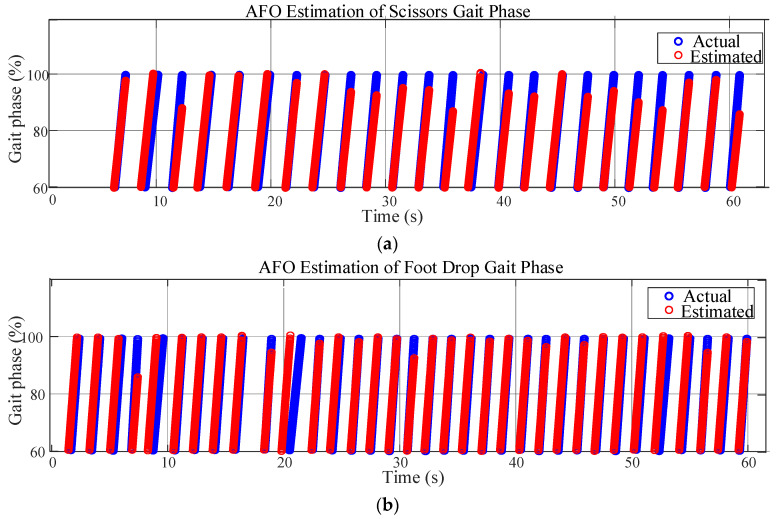

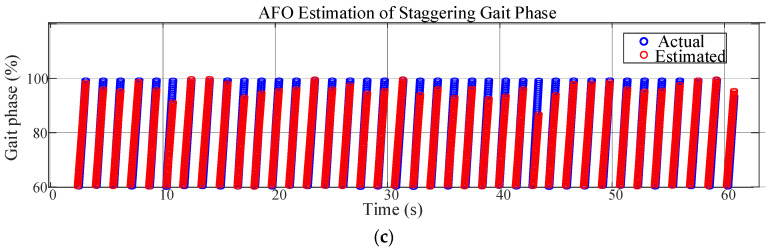
The AFO for phase estimation of abnormal gaits. Phase estimation of the (**a**) scissors gait, (**b**) foot-drop gait, and (**c**) staggering gait.

In this study, the left toe-off is taken as the initial phase. When using an AFO to estimate the phase, a mismatch between the left toe-off and phase 0 may occur as continuous estimation proceeds. Each time the left toe-off is detected, the estimation result of the AFO is forced to be reset to 0, thereby avoiding the mismatch between the event and the phase. The results of estimating three types of abnormal gaits using the AFO are shown in Figure 6.

In some periods of the scissors gait, there are relatively obvious deviations between the estimated phase and the actual phase. This may be because the characteristics of the scissors gait are complex (the movement pattern of legs crossing each other), which increases the difficulty of estimation. However, the overall trend remains synchronized, reflecting that the AFO has a certain adaptability to this gait. The fit between the estimated phase and the actual phase of the foot-drop gait is relatively high. The possible reason is that the characteristics of the foot-drop gait are relatively regular, and the AFO can identify and estimate them well, indicating that for such gaits with clear characteristics, the AFO algorithm performs better. For the staggering gait, the coincidence degree between the estimated phase and the actual phase is high in most periods. Although the staggering gait is unstable, the phase change has its own rhythm, and the AFO can effectively track it.

In conclusion, the estimated values of the AFO for the phases of the three abnormal gaits have a good overall follow-up with the actual phases, which indicates that the AFO is effective in estimating the phases of different abnormal gaits, and it can capture the change rules of the gait phases. The AFO is feasible for estimating the phases of the three abnormal gaits.

## 4. Lower Limb Movement Prediction Model Based on Gait Phases

With the aim of predicting lower limb movements (specifically the Euler angles of the thighs and shanks), the following two hypotheses were proposed in this study:

**Hypothesis 1 (H1).** 
*The motion signals of the ankle joints mainly reflect spatial position-related information, such as angles and displacements at specific moments. While gait phases precisely embody the temporal characteristics of the walking process. The effective integration of the spatial features contained in ankle joint motion signals and the temporal features represented by gait phases can more comprehensively depict the essential laws of human motion, thereby providing more sufficient information support for the accurate prediction of motion.*

**Hypothesis 2 (H2).** 
*The hybrid model combining the CNN and the LSTM (CNN_LSTM) possesses both the capability of the CNNs in extracting spatial features and the ability of the LSTMs in analyzing temporal signals, making it a more powerful model for motion prediction.*

**Figure 7:**
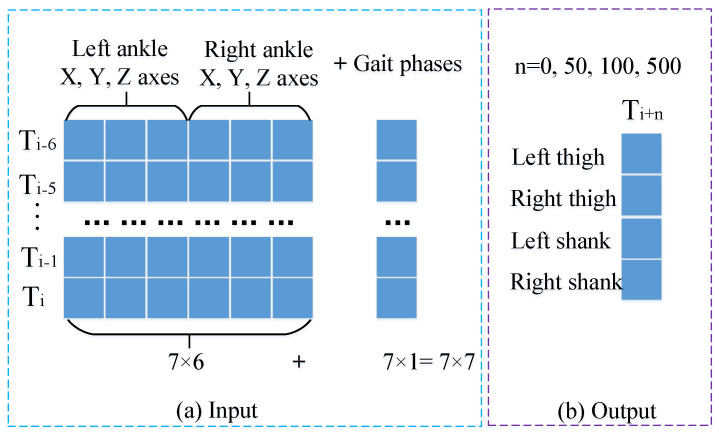
Input and output of the prediction model. (**a**) The feature-level fusion process of joint angles and phase information. For the current timestep, a 7 × 6 matrix was constructed using 6-axis ankle Euler angles (3 axes per ankle) from seven consecutive forward timesteps, while the corresponding phase values estimated by the AFO form a 7 × 1 matrix. These temporally aligned matrices were concatenated to generate a 7 × 7 composite input matrix for the joint angle prediction model. (**b**) The output structure of the prediction model: a 4 × 1 matrix constituted by the sagittal-plane Euler angles of the left and right thighs and left and right shanks at the predicted time instants. Specifically, the predicted time instants in this study correspond to the current moment, as well as 0.5 s, 1 s, and 5 s thereafter, respectively.

To verify H1, the CNN_LSTM model was employed as the prediction model. The prediction model was tested using two input modes—one combining ankle joint Euler angles with phases (angle-phase-integrated) and the other using ankle joint Euler angles without phases—to conduct comparative experiments on the prediction of lower limb movement angles. This was conducted to verify the effectiveness of the two prediction results. The angle-phase-integrated mode was in the form of a 7 × 7 matrix. This matrix was constructed by concatenating the three-axis Euler angles of the left and right ankles at seven consecutive time instants, as well as the gait phase estimation values at each instant. In the mode without combining phases, the input was only a 7 × 6 matrix formed by the three-axis Euler angles of the left and right ankles at seven consecutive time instants (denoted as CNN_LSTM76 hereinafter). The output is a 4 × 1 matrix of Euler angles for the left and right thighs and shanks in the sagittal plane at the prediction time point. Figure 7 illustrates both input configurations and the output structure.

**Figure 8:**
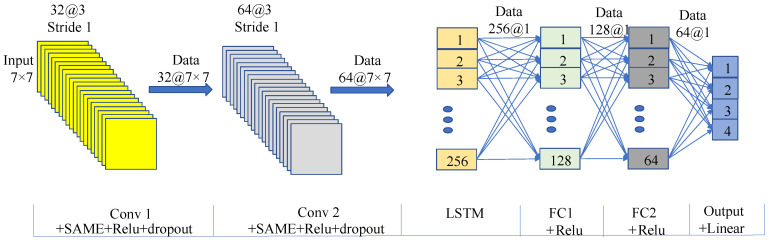
The structure of the CNN_LSTM for predicting lower limb angles. Other attributes of the CNN_LSTM network include mean squared error (MSE) as the loss function, 100 training epochs, and a learning rate of 0.001.

To verify H2, this study designs comparative experiments involving the CNN_LSTM, CNN, and LSTM networks. The structure of the CNN_LSTM model is shown in Figure 8.

**Figure 9:**
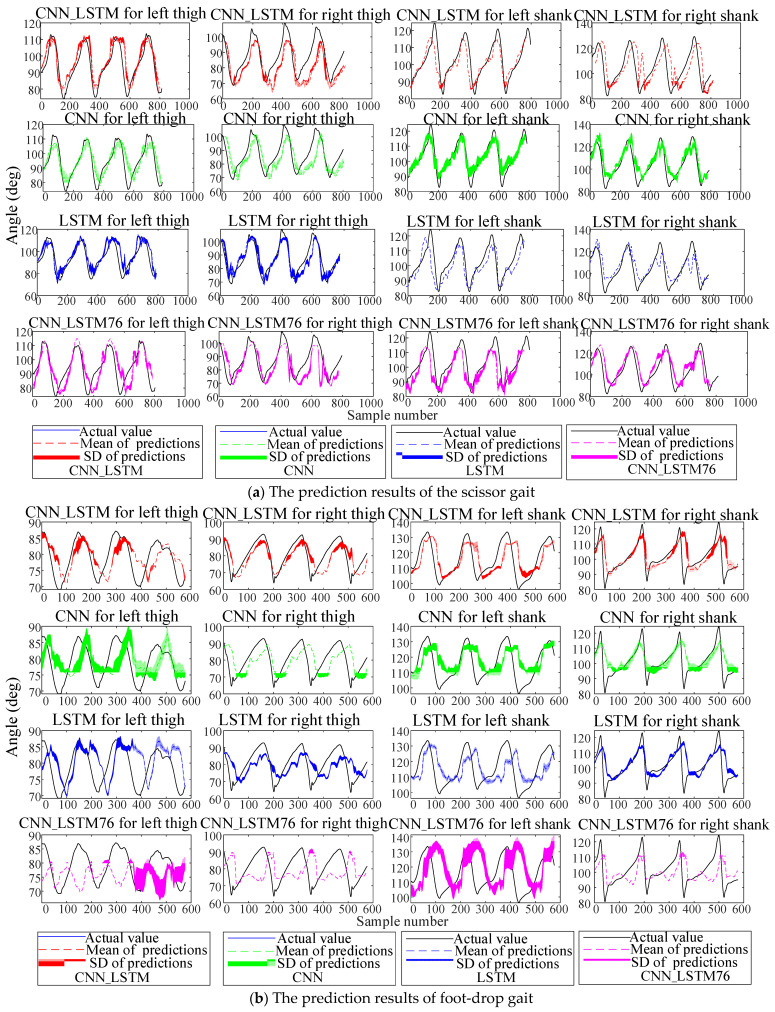

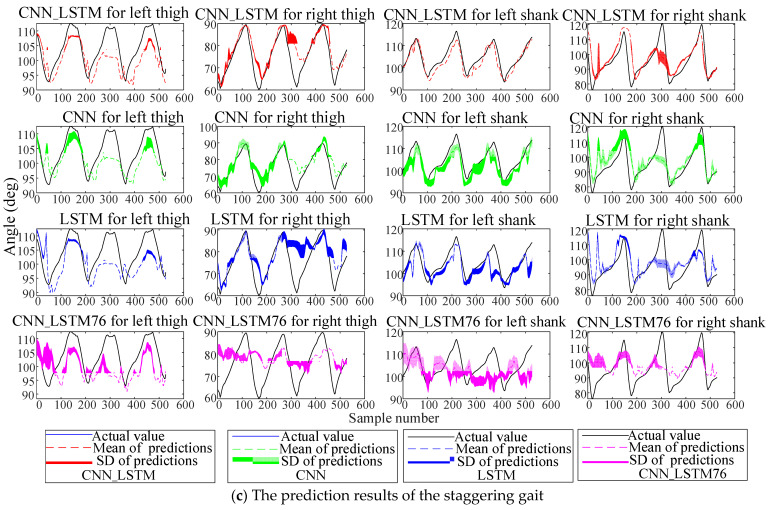
Predictions of the abnormal gait under four schemes. Solid lines represent actual values, dashed lines denote mean predictions, and shadows indicate prediction standard deviations.

The comparative CNN model removes the LSTM layers while maintaining identical structures and parameters to the CNN_LSTM. The comparative LSTM model removes the convolutional layers with other configurations consistent with the CNN_LSTM. Datasets were split into training, validation, and test sets at a 6:2:2 ratio, and each classifier underwent five independent experiments. Here, the prediction results of the four schemes (CNN_LSTM, CNN, LSTM, CNN_LSTM76) on the test set at 0.5 s are shown in Figure 9.

The results show that due to the left leg’s abnormal gait, the accuracy and stability of lower-limb angle prediction are lower than those of the right leg, reflecting the prediction difficulty under abnormal gaits. Notably, the CNN_LSTM outperformed other schemes in all five experiments. To quantitatively compare model performance, three metrics were adopted: root mean square error (RMSE), Pearson correlation coefficient (PCC), and total prediction time for training samples. Assuming the actual lower-limb angles are $\theta_{R}=\{\theta_{r,1},\theta_{r,2},\ldots ,\theta_{r,n}\}$ and predicted values are $\theta_{P}=\{\theta_{p,1},\theta_{p,2},\ldots ,\theta_{p,n}\}$, the RMSE and the PCC are defined as Equations (9) and (10):

(9) $$RMSE=\sqrt{\frac{1}{n}\sum_{i=1}^{n}{\left(\theta_{r,i}-\theta_{p,i}\right)}^{2}}$$

(10) $$PCC=\frac{Cov\left(\theta_{R},\theta_{P}\right)}{S_{\theta_{R}}S_{\theta_{P}}}$$

where $Cov\left(\theta_{R},\theta_{P}\right)$ is the covariance between the actual and predicted values, $S_{\theta_{R}}$ is the standard deviation of the actual values, and $S_{\theta_{P}}$ is the standard deviation of the predicted values. The *RMSE* reflects the degree of deviation between the predicted and actual values—smaller values indicate more stable prediction results across independent experiments. The *PCC* reflects the similarity between predicted and actual values, ranging from 0 to 1—larger values indicate closer correspondence. The *RMSE* and *PCC* of the test sets, as accuracy metrics, reflect the accuracy of the prediction model, while the total prediction time reflects the computational efficiency of the prediction model. Table 2, Table 3, Table 4 and Table 5 list the means and standard deviations of various evaluation metrics for each scheme across five experiments conducted at four prediction time points (current time, 0.5 s, 1 s, and 5 s later).

To verify statistical differences in prediction performance (measured by PCC), the Kruskal–Wallis test was first applied to assess overall group differences. Significant results were further analyzed with Dunn’s post-hoc test for pairwise comparisons. The predictions of the three angle-phase-integrated methods showed significantly better accuracy metrics than the CNN_LSTM76 (*p* < 0.0001). The statistical tests also showed that there was a statistical difference between the CNN–LSTM and the CNN (*p* = 0.0370); there was a statistical difference between the CNN–LSTM and the LSTM (*p* < 0.0001); and there was a statistical difference between the CNN and the LSTM (*p* < 0.0156). In terms of computational efficiency, there is no significant difference among the prediction models.

## 5. Discussion

### 5.1. Gait Phases

The classic Rancho Los Amigos phase classification aims to fully describe the kinematic characteristics of limb movements throughout the gait cycle. It typically subdivides the stance and swing phases into multiple subphases to capture key biomechanical processes, such as center of mass transfer and limb motion. While this approach comprehensively reflects the details of normal gait, it has two limitations when applied to the abnormal gait analysis and the LLE control as follows: First, overly refined stance subphases offer no practical value for designing assistive strategies (as the LLEs generally require no active actuation during the stance phase). Second, it struggles to adapt to the asynchronous movements and disordered phase characteristics of the left and right limbs in unilateral abnormal gait.

The four-phase classification proposed in this study differs fundamentally from existing schemes by focusing on the needs of the LLE assistance, with specific distinctions as follows:

- Task-oriented logic of division: The four-phase division is centered on “LLE assistive strategies”—since active assistance from the LLE only occurs during swing-related phases (pre-swing, swing, and swing termination), and no actuation is needed during the stance phase, there is no need to further subdivide the stance phase (retained only as “stance phase 0”). Instead, the emphasis is placed on distinguishing three swing subphases (1, 2, and 3) directly related to assistive actions. This reduces redundant phase information, directly linking phase detection to assistive control strategies and enhancing the specificity of LLE responses (e.g., “actuation preparation” in pre-swing phase 1, “active assistance” in swing phase 2, and “reset” in swing termination phase 3, each corresponding to distinct control commands).
- Coupled design of bilateral limb phases: Existing studies often detect single-leg phases independently [2,3,4], requiring subsequent calculations to derive the coordination between left and right limbs (e.g., based on assumptions of gait cycle synchronization). In our study, by defining “four phases” (combinations of left and right foot phases), the coupling relationship between bilateral limbs is directly embedded into the phase classification. For example, pre-swing phase 1 on one side is necessarily synchronized with swing termination phase 3 on the contralateral side, and swing phase 2 inherently corresponds to the contralateral stance phase 0. This design allows unilateral phases to be uniquely determined by contralateral phases (e.g., detecting the phase of the healthy side enables inference of the phase on the affected side), significantly improving the robustness of phase detection in unilateral abnormal gait—an advantage that existing single-leg-centered classification schemes cannot achieve. This constitutes an innovation of the present study.
- Simplicity and specificity of feature extraction: Existing studies often fuse multi-source data (such as plantar pressure, electromyographic signals, and multi-joint angles) to distinguish refined phases [22], whereas this study achieves phase discrimination based solely on trend changes in the sagittal-plane Euler angles of the ankle (a significant decrease in pre-swing phase 1 and a significant increase in swing phase 2). This feature selection simplifies sensor deployment (requiring only angle signals from IMUs) while specifically capturing the core motion characteristics of swing-related phases, avoiding feature redundancy caused by the need for phase refinement and better adapting to the lightweight, real-time application scenarios of the LLEs. Some studies have also deployed IMUs on the foot, with varied signal selections—some using angles [23], others gyroscope and acceleration data [24], and quaternions representing a potential alternative. Currently, there is no consensus on the optimal motion signal for the IMUs, warranting further investigation.

The discrete phase classification scheme, constructed in this study, is not merely an isolated tool for gait state recognition but serves as a critical bridge connecting the discrete features of human movement to continuous dynamic modeling, ultimately supporting the accurate estimation of continuous phases by the AFO. These discretized phase labels provide key event constraints for the AFO—enabling the oscillator to dynamically calibrate its periodic parameters based on known phase boundaries, effectively suppressing phase divergence in abnormal gait.

The gait phase classification was compared using the CNN and SVM models. Results showed that while the SVM performed slightly better in recognition speed, the CNN exhibited significantly superior classification performance. Thus, considering overall performance, the CNN is more suitable for gait phase classification.

Building on discrete gait phase recognition, this study conducted continuous phase estimation. Traditional continuous phase estimation employs the TBE, which typically extrapolates future continuous phases based on prior knowledge. However, when gait exhibits dynamic changes (e.g., cycle fluctuations due to speed adjustments), the TBE struggles to accurately match the gait cycle. Abnormal gait is often accompanied by a cycle disorder, irregular movement amplitudes, and poor rhythmic stability, making the TBE inadequate for precise continuous phase estimation in such cases. In contrast to the TBE, which relies on historical experience, an AFO can perceive real-time gait dynamics (such as joint movement speed and ground reaction force feedback) and dynamically adjust its oscillation frequency and phase parameters, aligning the phase estimation process closely with the real-time patterns of biological gait. AFOs thus provide a more biomechanically compatible solution for continuous phase estimation. The results of continuous phase estimation in this study confirm that the AFO is appropriate for abnormal gait. However, experiments also indicated that they perform poorly in scenarios with abrupt cadence changes (e.g., transitioning from standing to walking).

### 5.2. Prediction of Lower Limb Movement Angles

An angle-phase-integrated input mode was proposed in this study, which achieved accurate prediction of lower-limb angles at future moments by exploring the dynamic correlation between angles and phases, and this is another innovation of this study. The three prediction models, namely CNN_LSTM, CNN, and LSTM, adopted the angle-phase-integrated mode, while CNN_LSTM76 only took angles as input, and a comparative experiment was conducted among the four models. The statistical tests showed that the three prediction models with the angle-phase-integrated input mode have advantages over CNN_LSTM76, confirming that introducing gait phases based on ankle motion signals improves lower-limb prediction accuracy. H1 is, therefore, supported. Among the angle-phase-integrated modes, the CNN_LSTM is the optimal model for predicting lower-limb motion angles. Moreover, the CNN has better prediction performance than the LSTM. H2 is, therefore, verified.

For achieving effective control of the LLEs, it is essential to select the prediction point reasonably. If the prediction time point is chosen too close, there will not be sufficient time for sensor detection, gait phase classification, continuous phase estimation, lower-limb motion prediction, and actuator execution to respond. However, the prediction time point should not be chosen too far ahead either. Table 2, Table 3, Table 4 and Table 5 indicate that the prediction accuracy shows a decreasing trend as the prediction time point is extended. Choosing a prediction time point too far is not conducive to the precise control of the LLE.

In the algorithm design for gait classification and lower-limb motion angle prediction in this study, multiple lightweight optimization strategies were adopted to effectively reduce the computational load: At the sensor level, only the IMU was used, and only its angle signals were extracted as input, which greatly simplified the complexity of raw data. At the algorithm level, the redundant convolutional layers and pooling layers in the neural network were removed to strictly control the number of parameters of the classification and prediction models, thereby significantly improving the computational efficiency while ensuring the accuracy of core functions.

Through the analysis of the total end-to-end delay time, the time for signal detection and preprocessing is 0.010–0.050 s, the time for controller planning and decision making is 0.005–0.050 s, and the time for actuator execution is 0.010–0.100 s. The total end-to-end delay of the LLE is usually 0.200 s. Considering that the system should leave a certain redundancy, it is reasonable to select 0.500 s as the prediction point under the framework of this study, taking both real-time performance and control accuracy into comprehensive account.

### 5.3. Limitations

In the design of wearing and repeated experiments for the LLE in this study, we have fully considered the limitations in physical tolerance and cooperation ability of patients in pathological states, who would find it difficult to complete high-intensity and multiple rounds of experimental operations. Moreover, frequent wearing of an LLE may cause discomfort or carry a risk of secondary injury. Therefore, the abnormal gait data used in the current study were mainly obtained from healthy subjects, simulating three typical pathological gaits.

From the preliminary results of experimental verification, the abnormal gait data simulated by healthy subjects can, to a certain extent, reflect the adaptability of the proposed control approach in this study to abnormal walking patterns. Through the simulated data, we have verified the feasibility of the system in core functions such as gait phase recognition and lower-limb motion angle prediction. However, from the perspective of actual clinical application scenarios, the current data sources still have obvious limitations. There are essential differences between the abnormal gaits simulated by healthy subjects and real pathological gaits, which may lead to deviations between the experimental results and actual clinical needs. Hence, incorporating real pathological gait data will be a core focus of future work.

The current study only covers three typical abnormal gaits, while many other types prevalent in clinical practice are not involved. These gaits have their own particularities in movement patterns and biomechanical characteristics, and targeted research is needed for all of them. On one hand, it is necessary to expand the sample coverage of abnormal gaits and improve the diversity of the database. On the other hand, it is essential to analyze the core needs of different types of abnormal gaits and optimize the control strategy of the LLEs accordingly, so that the devices can better conform to the laws of physiological movement.

## 6. Conclusions

This study focuses on lower-limb gait recognition and motion angle prediction in the LLE research. A new gait phase division scheme was proposed for abnormal gaits, classifying single-leg gait into four discrete phases: pre-swing phase 1, swing phase 2, swing termination phase 3, and stance phase 0. The four phases of both legs form four walking stages: Gait 20, Gait 31, Gait 02, and Gait 13. This scheme is adaptable to both normal and abnormal gait detection. Three types of abnormal gaits were examined in this study: scissors, foot-drop, and staggering. Using sagittal ankle Euler angles as input, a CNN outperformed an SVM in discrete phase recognition for the three abnormal gaits. Further, a continuous phase estimation method based on the AFO was proposed, which exhibited good performance under abnormal gaits.

For lower-limb joint angle prediction, a CNN_LSTM model integrating gait phases and ankle motion signals was validated through comparative experiments with a CNN, an LSTM, and a phase-free CNN_LSTM76, with predictions made for four time points. The results showed that the input mode combining angle and phase significantly improves prediction accuracy, and the CNN_LSTM model is the optimal prediction model in this study.

In the next step, this study will establish human-exoskeleton coupled kinematic and dynamic models based on lower-limb angle prediction to realize the position and torque control of the LLE. From a longer-term perspective, future work should gradually narrow the gap between experimental research and clinical practice, promote the translation of the LLE technology from laboratory verification to real clinical application, and provide safe and effective movement assistance for patients with a wider range of gait disorders.

## Figures and Tables

**Table 1:** Comparison of the time consumption of the gait phase classifiers (Unit: s).

Classifier	Scissor Gait	Foot-Drop Gait	Staggering Gait
CNN	0.017–0.025	0.012–0.018	0.013–0.020
SVM	0.007–0.013	0.008–0.012	0.006–0.010

**Table 2:** Evaluation indicators of predictions for the current time by the four schemes.

Method	Scissor Gait			Foot-Drop Gait			Staggering Gait		
	RMSE (deg)	PCC	Time (s)	RMSE (deg)	PCC	Time (s)	RMSE (deg)	PCC	Time (s)
CNN_LSTM	2.7477 ± 0.0748	0.9794 ± 0.0011	0.084 ± 0.031	2.4886 ± 0.0654	0.9901 ± 0.0004	0.066 ± 0.017	3.8881 ± 0.0392	0.9632 ± 0.0010	0.086 ± 0.0289
CNN	3.8383 ± 0.3363	0.9609 ± 0.0080	0.083 ± 0.030	3.8179 ± 0.4300	0.9804 ± 0.0026	0.076 ± 0.029	3.8755 ± 0.5621	0.9669 ± 0.0081	0.069 ± 0.031
LSTM	4.9435 ± 0.3164	0.9344 ± 0.0068	0.104 ± 0.021	3.2400 ± 0.1396	0.9833 ± 0.0015	0.060 ± 0.010	4.6493 ± 0.1970	0.9454 ± 0.0036	0.089 ± 0.014
CNN_LSTM76	4.5832 ± 0.3375	0.9440 ± 0.0093	0.082 ± 0.015	6.3669 ± 0.2530	0.9347 ± 0.0063	0.077 ± 0.007	6.5683 ± 0.4681	0.8901 ± 0.0131	0.089 ± 0.024

**Table 3:** Evaluation indicators of predictions for 0.5 s later by the four schemes.

Method	Scissor Gait			Foot-Drop Gait			Staggering Gait		
	RMSE (deg)	PCC	Time (s)	RMSE (deg)	PCC	Time (s)	RMSE (deg)	PCC	Time (s)
CNN_LSTM	3.5703 ± 0.1254	0.9663 ± 0.0025	0.085 ± 0.016	4.5216 ± 0.1651	0.9702 ± 0.0016	0.072 ± 0.014	4.9525 ± 0.1108	0.9351 ± 0.0033	0.096 ± 0.024
CNN	4.4740 ± 0.2990	0.9445 ± 0.0082	0.087 ± 0.023	5.1143 ± 0.3699	0.9621 ± 0.0051	0.094 ± 0.042	5.6524 ± 0.3038	0.9196 ± 0.0097	0.0978 ± 0.033
LSTM	5.0903 ± 0.1094	0.9282 ± 0.0027	0.084 ± 0.014	5.4372 ± 0.3944	0.9574 ± 0.0049	0.073 ± 0.013	5.9591 ± 0.1199	0.9067 ± 0.0037	0.081 ± 0.020
CNN_LSTM76	5.4911 ± 0.3296	0.9156 ± 0.0114	0.080 ± 0.012	7.6056 ± 0.3590	0.9056 ± 0.0057	0.065 ± 0.012	6.9296 ± 0.2409	0.8714 ± 0.0075	0.091 ± 0.017

**Table 4:** Evaluation indicators of predictions for 1 s later by the four schemes.

Method	Scissor Gait			Foot-Drop Gait			Staggering Gait		
	RMSE (deg)	PCC	Time (s)	RMSE (deg)	PCC	Time (s)	RMSE (deg)	PCC	Time (s)
CNN_LSTM	4.0357 ± 0.0970	0.9547 ± 0.0022	0.082 ± 0.023	4.4899 ± 0.0511	0.9672 ± 0.0008	0.066 ± 0.014	4.1708 ± 0.0776	0.9563 ± 0.0017	0.064 ± 0.013
CNN	5.2890 ± 0.5285	0.9216 ± 0.0169	0.074 ± 0.015	5.2492 ± 0.1635	0.9567 ± 0.0023	0.078 ± 0.014	4.9307 ± 0.3629	0.9445 ± 0.0056	0.070 ± 0.027
LSTM	6.0366 ± 0.6660	0.8968 ± 0.0194	0.089 ± 0.018	4.7741 ± 0.0676	0.9619 ± 0.0010	0.069 ± 0.016	5.3619 ± 0.1940	0.9287 ± 0.0087	0.062 ± 0.001
CNN_LSTM76	6.1589 ± 0.3075	0.8916 ± 0.0113	0.067 ± 0.015	8.2071 ± 0.6281	0.8916 ± 0.0201	0.055 ± 0.009	8.1783 ± 0.3064	0.8243 ± 0.0155	0.047 ± 0.001

**Table 5:** Evaluation indicators of predictions for 5 s later by the four schemes.

Method	Scissor Gait			Foot-Drop Gait			Staggering Gait		
	RMSE (deg)	PCC	Time (s)	RMSE (deg)	PCC	Time (s)	RMSE (deg)	PCC	Time (s)
CNN_LSTM	5.9570 ± 0.2599	0.9025 ± 0.0079	0.067 ± 0.010	7.2559 ± 0.2573	0.9150 ± 0.0058	0.066 ± 0.020	6.0462 ± 0.4199	0.9070 ± 0.0148	0.078 ± 0.026
CNN	6.3811 ± 0.4805	0.8856 ± 0.0166	0.062 ± 0.004	8.3599 ± 0.3619	0.8922 ± 0.0116	0.064 ± 0.011	5.9421 ± 0.4922	0.9151 ± 0.0181	0.084 ± 0.041
LSTM	6.7458 ± 0.2473	0.8781 ± 0.0071	0.064 ± 0.010	8.6085 ± 0.1091	0.8768 ± 0.0035	0.068 ± 0.006	7.0929 ± 0.3090	0.8720 ± 0.0125	0.068 ± 0.017
CNN_LSTM76	9.1759 ± 0.1880	0.7568 ± 0.0082	0.080 ± 0.008	12.0965 ± 0.7693	0.7926 ± 0.0194	0.061 ± 0.008	8.4411 ± 0.4004	0.8104 ± 0.0219	0.086 ± 0.030

## Data Availability

The dataset is available on request from the authors.

## Abbreviations

The following abbreviations were used in this manuscript:

AFO	Adaptive Frequency Oscillator
CNN	Convolutional Neural Network
CPG	Central Pattern Generator
DMP	Dynamic Movement Primitives
EKF	Extended Kalman Filter
HMI	Human-Machine Interaction
LLE	Lower-Limb Exoskeleton
LSTM	Long Short-Term Memory Network
PCC	Pearson Correlation Coefficient
RMSE	Root Mean Square Error
SVM	Support Vector Machine
